# A Low-Ambiguity Signal Waveform for Pseudolite Positioning Systems Based on Chirp

**DOI:** 10.3390/s18051326

**Published:** 2018-04-25

**Authors:** Qing Liu, Zhigang Huang, Yanhong Kou, Jinling Wang

**Affiliations:** 1 School of Electronic and Information Engineering, Beihang University, Beijing 100083, China; baahzg@163.com (Z.H.); kouy@buaa.edu.cn (Y.K.); 2 School of Civil and Environmental Engineering, University of New South Wales, Sydney, NSW 2052, Australia; jinling.wang@unsw.edu.au

**Keywords:** chirp, low-ambiguity, high-precision, navigation, pseudolite

## Abstract

Signal modulation is an essential design factor of a positioning system, which directly impacts the system’s potential performance. Chirp compressions have been widely applied in the fields of communication, radar, and indoor positioning owing to their high compression gain and good resistance to narrowband interferences and multipath fading. Based on linear chirp, we present a modulation method named chirped pseudo-noise (ChPN). The mathematical model of the ChPN signal is provided with its auto-correlation function (ACF) and the power spectrum density (PSD) derived. The ChPN with orthogonal chirps is also discussed, which has better resistance to near-far effect. Then the generation and detection methods as well as the performances of ChPN are discussed by theoretical analysis and simulation. The results show that, for ChPN signals with the same main-lobe bandwidth (MLB), generally, the signal with a larger sweep bandwidth has better tracking precision and multipath resistance. ChPN yields slighter ACF peaks ambiguity due to its lower ACF side-peaks, although its tracking precision is a little worse than that of a binary offset carrier (BOC) with the same MLB. Moreover, ChPN provides better overall anti-multipath performance than BOC. For the ChPN signals with the same code rate, a signal with a larger sweep bandwidth has better performance in most aspects. In engineering practice, a ChPN receiver can be implemented by minor modifications of a BOC receiver. Thus, ChPN modulation shows promise for future positioning applications.

## 1. Introduction

Global Navigation Satellite System (GNSS) positioning accuracy degrades significantly in indoor and urban canyon environments due to severe signal attenuations and multipath effects, which has been motivating researchers to explore alternative technologies for location-based service (LBS) in recent years [1,2,3,4]. GNSS-like positioning technologies, for example, pseudolite positioning systems (PLs) and GNSS repeater systems, have been extensively investigated for indoor and urban applications [5,6,7]. A pseudolite system employs signals the same as or similar to GPS signals for ranging [8,9]. As an essential design consideration of a positioning system, the signal modulation method directly affects the inherent performance of the system [10].

GNSS-like technologies are widely investigated for positioning in GNSS signal blocked situations, e.g., indoor, urban canyons, and open-pit mines, etc. It is well known that high accuracy and flexibility are the basic requirements for such applications. The portable transmitters, e.g., PLs powered by batteries, and receivers have become more and more significant, which made us to explore low-power consumption solutions. BPSK signals with a larger bandwidth can provide better tracking precision, but require correspondingly greater transmitter power to conserve integrated energy per code epoch [11]. High order BOC signals may provide high tracking performance without shortening the chip width so that will not increase the power consumption. The large side-peaks of its ACF (auto-correlation function), however, may lead to false locking. Therefore, it is of practical significance to design a modulation method to meet the requirements.

Chirp signal sweeps over a frequency band in a certain time duration, with which one can spread the spectrum without shrinking the chip width, so that it will not increase the power consumption compared with BPSK and quite suitable for a GNSS-like system which is low data-rate. Recent advances in surface acoustic wave (SAW) devices have shown that chirp generators and matched filters can be implemented by using completely passive, low-cost SAW chirp delay lines [12]. Nanotron Technologies (Berlin, Germany) has brought forth some mature chirp integrated circuits (IC) at a price of about 40 Euro per chip, that work in the industrial, scientific and medical (ISM) radio band. In addition, SEMTECH (Camarillo, CA, USA) and SAGE Millimeter (Torrance, CA, USA) have also developed chirp products that work in various radio bands.

Chirp signals have been widely used in radar systems [13,14,15]. Owing to their properties of high compression gain, good resistance to narrowband interferences and multipath effects [12,14], chirp signals have also been used extensively in other fields, such as measurement [16], ultra-wide band communication [17] and optical fiber communication [12]. Furthermore, the chirp signal is also introduced for positioning and navigation. Typically, Nanotron has presented an positioning system based on CSS (chirp spread spectrum, a globally patented design of the company), and successfully used in open-pit mining, and its positioning products, nanoLOCs, are widely used in indoor positioning [18]. What’s more, [19] employs chirp in an RFID indoor positioning system, some experiments also have been done by researchers and obtained some useful results [18,20,21]. Another signal named absolute phase modulation (APM), which is based on non-linear chirp, has also been introduced to positioning systems [22]. Most chirp-based positioning systems are two-way ranging systems. They have several shortcomings, such as limited system capacity and the need for more base-stations, compared with one-way ranging systems.

Based on the linear chirp technique, we present a modulation method named chirped pseudo-noise (ChPN). Its principle is similar to BOC modulation by replacing the BOC sub-carrier with one period of chirp. As will be demonstrated later, ChPN inherits the attractive features of both PRN code and chirp signals, such as excellent auto-correlation and cross-correlation properties, fine multipath resolution, and high compression gain. Our contributions are as follows: (1) the signal model as well as ACF and PSD are derived; (2) the signal performances are evaluated by theoretical analysis and simulation; (3) the ChPN with orthogonal chirps is also discussed. Since the ACF side-peaks of the ChPN signal are much lower than that of the BOC modulation, the wrong acquisition and false lock problems caused by the ACF peak ambiguity can be mitigated. Although the slightly sharper ACF main peak of the BOC signal promises a little higher tracking precision, the multipath-induced tracking biases converge much slower than the ChPN signal with the multipath delay increasing. The near-far effect can be mitigated by the ChPNs with orthogonal chirps. However, its candidates amount is subject to signal bandwidth. From the perspective of receiver complexity, the ChPN receivers can be implemented by a slight modification of the BOC receiver. Thus, ChPN modulation provides a good candidate for pseudolite ranging signal design.

The remainder of the paper is organized as follows: the time-domain model of the ChPN signal will be provided in Section 2, followed by its ACF and power spectrum density (PSD). Section 3 will analyze the generation and detection methods of the ChPN signal. Section 4 will provide a theoretical analysis for signal performance. The simulation is done in the Section 5. Conclusions will be given in Section 6. Finally, Appendix A gives the detailed derivation of the chirp spectrum.

## 2. Mathematical Models

The time-domain model, ACF, and PSD expressions of ChPN will be explored in this section.

### 2.1. Modulation Principle

**Figure 1:**
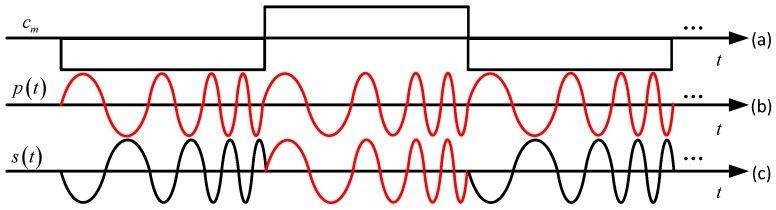
Illustration of waveforms of (**a**) PRN code signal; (**b**) Chirp signal; (**c**) ChPN signal.

The ChPN signal can be expressed as the following equation:

(1) $$s\left(t\right)=\sum_{-\infty }^{\infty }c_{m}p\left(t-mT_{c}\right)$$

with the spread spectrum symbol waveform, p(t), as:

(2) $$p\left(t\right)=\sqrt{2}\cos\left(2\pi f_{c}t\pm \pi \frac{B_{s}}{T_{c}}t^{2}\right)g\left(\frac{t}{T_{c}}\right),\;-\frac{T_{c}}{2}<t<\frac{T_{c}}{2}$$

where $\left\{c_{m}=\pm 1\right\}$ is the PRN sequence, $T_{c}$ is the chip duration, $f_{c}$ is the center frequency of chirp, $B_{s}$ is the sweep bandwidth, $g\left(t/T_{c}\right)$ is the gate function with a duration of $T_{c}$. The amplitude of p(t) is assumed as $\sqrt{2}$ to ensure the power is 1, without loss of generality. A chirp with frequency increasing is called an up-chirp, otherwise is called a down-chirp [17]. While only up-chirp is taken as an example hereinafter, the techniques and conclusions in this paper are also applicable to the down-chirp case. The waveform of the ChPN signal is illustrated in Figure 1.

Henceforth, the notation ChPN(*p*,*q*) will denote a ChPN signal with a sweep bandwidth of $B_{s}=p\times f_{0}$ and a code rate of $q\times f_{0}$, respectively, with $f_{0}=1.023\;\mathrm{MHz}$. The main-lobe bandwidth (MLB) of the ChPN signal is determined by the chipping rate and the sweep range. As most signal energy is concentrated on the band $-\left(p+q\right)f_{0}$~$\left(p+q\right)f_{0}$, the MLB can be expressed as:

(3) $$B_{chp}=2\left(p+q\right)f_{0}$$

According to Equation (3), there exist various modulations with the same MLB if we change parameters *p* and *q*. It is necessary for us to make comparisons for them that will be carried out in Section 4 and Section 5.

### 2.2. Auto-Correlation Function

The ACF of the ChPN signal, R(t+τ,t), can be expressed as:

(4) $$R\left(t+\tau ,t\right)=E\left[s{\left(t\right)}^{*}s\left(t+\tau \right)\right]=\sum_{k=-\infty }^{+\infty }\sum_{n=-\infty }^{+\infty }\left\{\begin{array}{l} E\left(c_{n}{}^{*}c_{n+k}\right){\left[p\left(t-nT_{c}\right)\right]}^{*}\\ \times p\left(t-nT_{c}+\tau -kT_{c}\right) \end{array}\right\}$$

According to (4), “*” represents complex conjugate, R(t+τ,t) is a periodic function with respect to the variable t with a period of $T_{c}$; (1) indicates that the mean value of s(t) is also a periodic function with the same period $T_{c}$. So the ChPN signal is cyclostationary, and its ACF, R(τ) with t eliminated, can be obtained by the time average of R(t+τ,t):

(5) $$R\left(\tau \right)=\frac{1}{T_{c}}\sum_{k=-\infty }^{+\infty }R_{c}\left(k\right)R_{p}\left(\tau -kT_{c}\right)$$

$R_{p}\left(\tau \right)$ is the ACF of p(t), $R_{c}\left(k\right)$ is the ACF of $c_{n}$. Assuming that the PRN code sequence is random, non-periodic, identically distributed, equiprobable and independent, we have:

(6) $$R_{c}\left(k\right)=E\left({c_{n}}^{*}c_{n+k}\right)\approx \{\begin{array}{c} 1,k=0\\ 0,k\neq 0 \end{array}$$

Thus, (5) can be simplified as:

(7) $$R\left(\tau \right)=R_{p}\left(\tau \right)/T_{c}$$

where the $R_{p}\left(\tau \right)$ is as follows [23]:

(8) $$R_{p}\left(\tau \right)=T_{c}\frac{\sin\left(\pi B_{s}\tau \left(1-\frac{\left|\tau \right|}{T_{c}}\right)\right)}{\pi B_{s}\tau }\cos\left(2\pi f_{0}\tau \right),-T_{c}<\tau <T_{c}$$

Based on Equations (7) and (8), the ACF is given by:

(9) $$R\left(\tau \right)=\frac{\sin\left(\pi B_{s}\tau \left(1-\frac{\left|\tau \right|}{T_{c}}\right)\right)}{\pi B_{s}\tau }\cos\left(2\pi f_{c}\tau \right),\;-T_{c}<\tau <T_{c}$$

Based on the Equation (9), the computer numerical results show that the ACF curve reaches its maximum at τ=0 and its first zero at $\tau \approx \pm 1/2B_{s}$, and the ACF main peak width is about $1/B_{s}$. Therefore, the sharpness of the ACF main peak is proportional to $B_{s}$; a wide $B_{s}$ results in a sharp correlation peak, and thus a fine multipath resolution as well as good acquisition and tracking performance. What’s more, the signal-to-noise ratio (SNR) of the output of the match filter in the receiver is amplified by the following two factors: (1) the compression gain of chirp, $G_{c}=10\log\left(B_{s}T_{c}\right)$ dB [24]; (2) the dispreading gain $10\log\left(N_{c}\right)$ dB, where $N_{c}$ is the PRN code length.

### 2.3. Power Spectrum Density

The PSD can be derived from the following expression [25]:

(10) $$S_{chp}\left(f\right)=\frac{1}{T_{c}}{\left|P\left(f\right)\right|}^{2}S_{c}\left(f\right)$$

where, |P(f)| is the amplitude spectrum of p(t); $S_{c}\left(f\right)$ is the PSD of the PRN sequence $\left\{c_{m}\right\}$ as follows:

(11) $$S_{c}\left(f\right)=\sum_{k=-\infty }^{\infty }R_{c}\left(k\right)e^{-j2\pi fkT_{c}}=1$$

After a complicated derivation (the detailed derivation is shown in Appendix A), P(f) is:

(12) $$P\left(f\right)=\frac{1}{2}\sqrt{\frac{T}{B_{s}}}\left\{\begin{array}{l} \exp\left[-j\frac{T_{c}\pi {\left(f-f_{0}\right)}^{2}}{B_{s}}\right]\\ \times \left[C\left(X_{1}\right)+jS\left(X_{1}\right)+C\left(X_{2}\right)+jS\left(X_{2}\right)\right]\\ +\exp\left[j\frac{T_{c}\pi {\left(f+f_{0}\right)}^{2}}{B_{s}}\right]\\ \times \left[C\left(X_{3}\right)+C\left(X_{4}\right)-jS\left(X_{3}\right)-jS\left(X_{4}\right)\right] \end{array}\right\}$$

where:

$$X_{1}=\frac{B_{s}+2\left(f-f_{c}\right)}{\sqrt{2B_{s}/T_{c}}},X_{2}=\frac{B_{s}-2\left(f-f_{c}\right)}{\sqrt{2B_{s}/T_{c}}}$$

$$X_{3}=\frac{B_{s}-2\left(f+f_{c}\right)}{\sqrt{2B_{s}/T_{c}}},X_{4}=\frac{B_{s}+2\left(f+f_{c}\right)}{\sqrt{2B_{s}/T_{c}}}$$

C(X) and S(X) are the Fresnel cosine and sine integrals, respectively. Simplifying (12) yields:

(13) $$P\left(f\right)=\sqrt{A^{2}+B^{2}+2AB\cos\left(\theta_{1}-\theta_{2}\right)}\exp\left(j\arctan\left(\frac{A\sin\left(\theta_{1}\right)+B\sin\left(\theta_{2}\right)}{A\cos\left(\theta_{1}\right)+B\cos\left(\theta_{2}\right)}\right)\right)$$

where:

$$A=\frac{1}{2}\sqrt{\frac{T_{c}}{B_{s}}}\sqrt{{\left(C\left(X_{1}\right)+C\left(X_{2}\right)\right)}^{2}+{\left(S\left(X_{1}\right)+S\left(X_{2}\right)\right)}^{2}}$$

$$B=\frac{1}{2}\sqrt{\frac{T_{c}}{B_{s}}}\sqrt{{\left(C\left(X_{3}\right)+C\left(X_{4}\right)\right)}^{2}+{\left(S\left(X_{3}\right)+S\left(X_{4}\right)\right)}^{2}}$$

$$\theta_{1}=-\frac{\pi T_{c}{\left(f-f_{0}\right)}^{2}}{B_{s}}+\arctan\left(\frac{S\left(X_{1}\right)+S\left(X_{2}\right)}{C\left(X_{1}\right)+C\left(X_{2}\right)}\right)$$

$$\theta_{2}=\frac{\pi T_{c}{\left(f+f_{0}\right)}^{2}}{B_{s}}-\arctan\left(\frac{S\left(X_{3}\right)+S\left(X_{4}\right)}{C\left(X_{3}\right)+C\left(X_{4}\right)}\right)$$

Substituting (11) and (13) into (10) yields the PSD expression:

(14) $$S_{chp}\left(f\right)=\left(A^{2}+B^{2}+2AB\cos\left(\theta_{1}-\theta_{2}\right)\right)/T_{c}$$

### 2.4. ChPN with Orthogonal Chirps

If the better cross-correlation performance is wanted, we can select the chirp waveforms based on the following equation [12]:

(15) $$p_{orth}\left(t\right)=\sqrt{2}\cos\left(\frac{\pi N}{T_{c}^{2}}{\left(t-k\frac{T_{c}}{N}\right)}^{2}-\frac{\pi }{4}\right)-\frac{T_{c}}{2}<t<\frac{T_{c}}{2}$$

where $N=B_{s}T_{c}$, and k=0, 1, …, N−1. Replacing p(t) with $p_{orth}\left(t\right)$ in Equation (1), then the ChPN with orthogonal chirps obtained, which is denoted as O-ChPN. Due to the constraint condition, the transmitter number is limited to *N*, i.e., the maximum number of transmitters is 5 with a $B_{s}$ of $5f_{0}$, and a $T_{c}$ of $1/f_{0}$. Both the chirp waveforms and PN sequences are different among the transmitters. The further discussion will be present in Section 4.5 and Section 5.

## 3. Signal Generation and Detection

Traditionally, a chirp signal can be generated with analog circuitry via a voltage-controlled oscillator (VCO). Recent advances in SAW devices have made it easier to generate chirp in a low-cost way. Chirp can also be generated digitally by using a direct digital synthesizer (DDS) [14].

**Figure 2:**
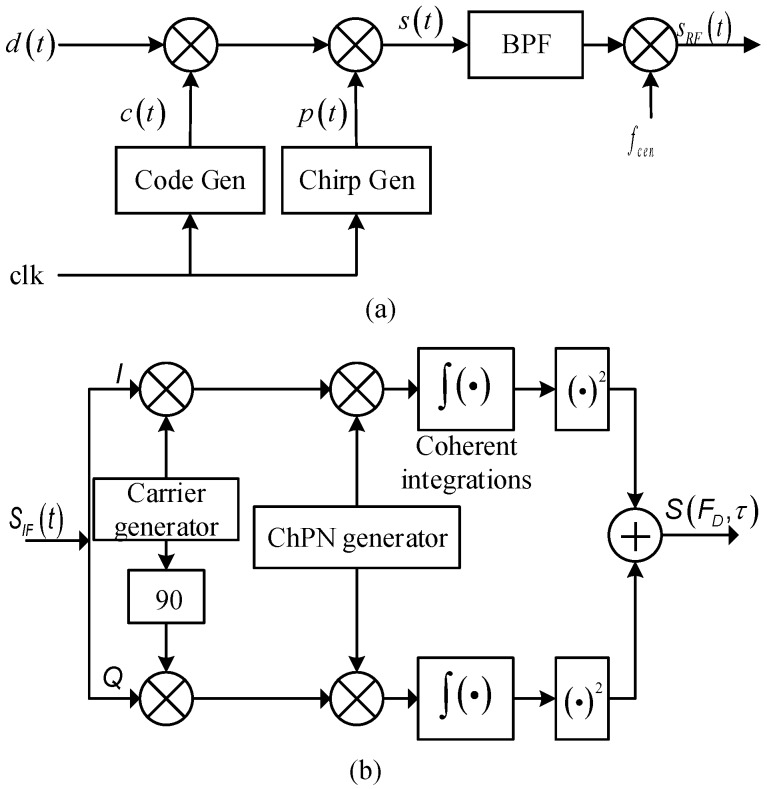
ChPN generating schematic diagram (**a**); Regular acquisition scheme of ChPN signal (**b**).

Since the modulation principle of ChPN is similar to BOC, the ChPN signal can be generated by replacing the BOC sub-carrier generating module with the chirp generating module, as shown in Figure 2a. In the figure, the data waveform d(t) is modulated by the PRN code signal c(t) and the chirp sub-carrier p(t) to form a baseband signal s(t). After passing through the band-pass filter (BPF) and being modulated by the radio frequency (RF) carrier, s(t) is up-converted to the RF signal $s_{RF}\left(t\right)$. The clock signals driving PRN code and chirp generation modules should be derived coherently from the same oscillator source.

A regular acquisition scheme of ChPN signals is shown in Figure 2b, which is similar to that of BOC signals. First, the RF signal $s_{RF}\left(t\right)$ was mixed with the local oscillator (LO) signal, down-converted to an intermediate frequency (IF); then, the IF signal $S_{IF}\left(t\right)$ is down-converted by the local carrier and cross-correlated by the local ChPN reference signal, respectively; next, after coherent integrations and square operations, the decision variable can be obtained by adding the outputs of I and Q channels as the following:

(16) $$\{\begin{array}{c} S_{I}=\sqrt{C/2}R\left(\tau \right)\cos\left(\theta \right)\mathrm{sinc}\left(\pi F_{D}T_{p}\right)+n_{I}\\ S_{Q}=\sqrt{C/2}R\left(\tau \right)\sin\left(\theta \right)\mathrm{sinc}\left(\pi F_{D}T_{p}\right)+n_{Q} \end{array}$$

where C is the signal power, τ is the delay difference between the local code and the incoming code, θ is the phase difference between the local carrier and the incoming signal carrier, $F_{D}$ is the frequency difference between the local carrier and the incoming signal carrier, $T_{p}$ is the pre-detection integration time, $n_{I}$ and $n_{Q}$ are the Gaussian noises in the correlator outputs. The decision variable $S\left(F_{D},\tau \right)$ can be expressed as:

(17) $$S\left(F_{D},\tau \right)=S_{I}{}^{2}+S_{Q}{}^{2}$$

which is a random process with a chi-square distribution and two degrees of freedom, and its non-centrality parameter is λ=C2sinc2(πFDTp)R2(τ).

## 4. Numerical Results

In this section, we will divide the discussion into five parts. First, ACF and PSD are analyzed as they are the most essential characteristics for a navigation signal. The second part will focus on the tracking precision, which is the most concerned in most civil applications. The multipath resistance will be discussed in the third part, as it is the main error source in a GNSS-like positioning system and can hardly be eliminated. Then, the tracking ambiguity is analyzed as the ACF contain multiple peaks. Finally, the cross-correlation comparison among several signal signals. Since ChPN(5,1) keeps the spectral characteristics of chirp with a relatively reasonable bandwidth, it is selected as the representative for the comparison between different signal waveforms. The analysis will be carried out in three situations as Table 1 shows.

### 4.1. ACF and PSD

**Figure 3:**
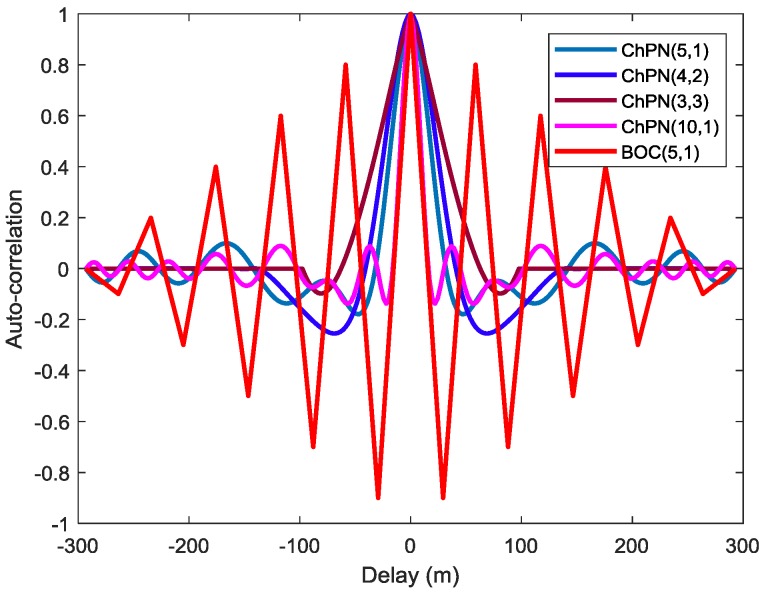
The auto-correlation of several modulations.

Figure 3 is drawn with infinite front-end bandwidth. For the first situation, as shown in that figure, the ChPN(5,1) has the sharpest ACF main peak among the alternative options, while ChPN(4,2) has a sharper ACF peak than ChPN(3,3) in a relative large time delay and a more obtuse ACF peak in a small time delay. Therefore, ChPN(5,1) may provide the best tracking precision, ChPN(4,2) may provide a better tracking precision than ChPN(3,3) with a small previous detection bandwidth (PDB), and worse tracking precision with a large PDB based on the tracking theory provided by Betz [26,27]. What’s more, ChPN(4,2) has the largest side-peak amplitude, while ChPN(3,3) has the smallest one. For the second situation, BOC(5,1) has sharper ACF main peak than ChPN(5,1), which may lead to better tracking precision. However, the ACF side-peaks of ChPN(5,1) are much lower than that of BOC(5,1), which yields a larger noise margin and thus a lower false locking probability if the traditional E-L discriminator is used for the delay lock loop (DLL). The larger main-peak to maximum-side-peak ratio (MSPR) can reflect the lower ambiguity. The MSPRs of ChPN(5,1) and BOC(5,1) are about 15 dB and 0.91 dB, respectively. Therefore, the ACF peak ambiguity problem can be mitigated by ChPN, which makes it easier to track the correct ACF peak using the traditional E-L discriminator. For the third situation, ChPN(10,1) has a shaper ACF main peak, and more ACF side-peaks, which may lead to better tracking precision and more serious ACF peaks ambiguity, respectively. Owing to the symmetry of the ACF, the “Bump and Jump” technique can be employed to achieve the unambiguous tracking of ChPN signal, which will increase the receiver complexity.

**Figure 4:**
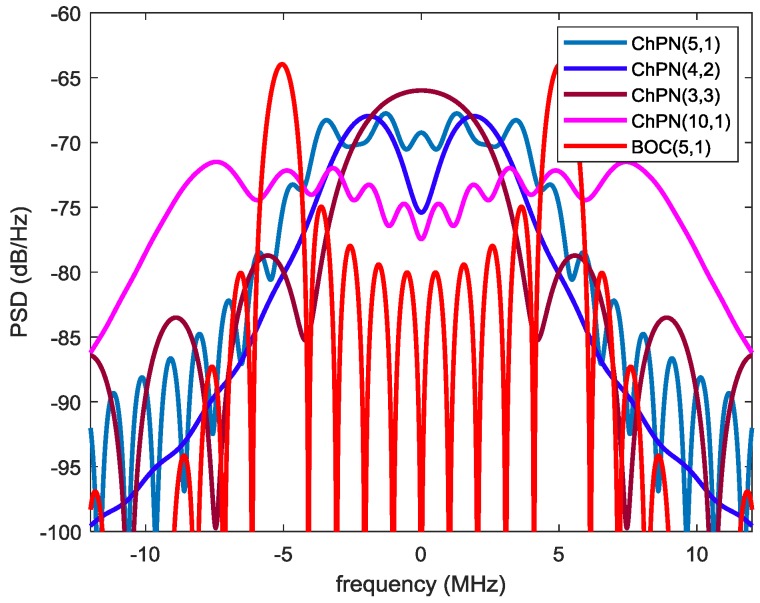
PSD envelopes of various signals.

According to (14), the PSD envelopes for ChPN(5,1), ChPN(4,2), ChPN(3,3) and ChPN(10,1), and BOC(5,1) are drawn in Figure 4. As we can see from the figure, some conclusions can be drawn. For the first situation, the PSD of ChPN(5,1) is flatter than the others as well as more high-frequency components, ChPN(4,2) owns more frequency components in the band of 1 MHz to 5 MHz while ChPN(3,3) owns more frequency components in band with higher absolute value of frequency. Hence, ChPN(3,3) may has a better tracking precision than ChPN(4,2) with a large PDB. For the second situation, the PSD of BOC(5,1) contains more high-frequency components than ChPN(5,1), which may yield a better tracking precision. For the third situation, ChPN(10,1) has a flatter PSD envelopes and contains more high-frequency components than ChPN(5,1), which may contribute to better tracking precision.

### 4.2. Tracking Performance

For the evaluation of the 1-sigma thermal noise jitter of DLL tracking, [28] developed a low bound of tracking error (LBTE), which is independent of specific code tracking circuit design and reflects the ultimate tracking performance. Considering the similarity of the modulation principle of ChPN with BOC signals, the canonical model of code-tracking loop can be employed to evaluate the ChPN tracking performance [26]. The LBTE of the time-of-arrival (TOA) estimate of this model can be written as:

(18) $$\sigma_{LB}≅\frac{1}{T_{c}}\sqrt{\frac{B_{L}\left(1-0.5B_{L}T\right)}{{\left(2\pi \right)}^{2}C\int_{-\beta_{r}/2}^{\beta_{r}/2}f^{2}\left[G_{s}\left(f\right)/G_{w}\left(f\right)\right]df}}\left(\mathrm{chip}\right)$$

where $B_{L}$ is the loop bandwidth, T is the coherent integration time, C is the received signal power, $\beta_{r}$ is the PDB, $G_{s}\left(f\right)$ is the normalized signal PSD, $G_{w}\left(f\right)$ is the PSD of the sum of noise plus interference.

**Figure 5:**
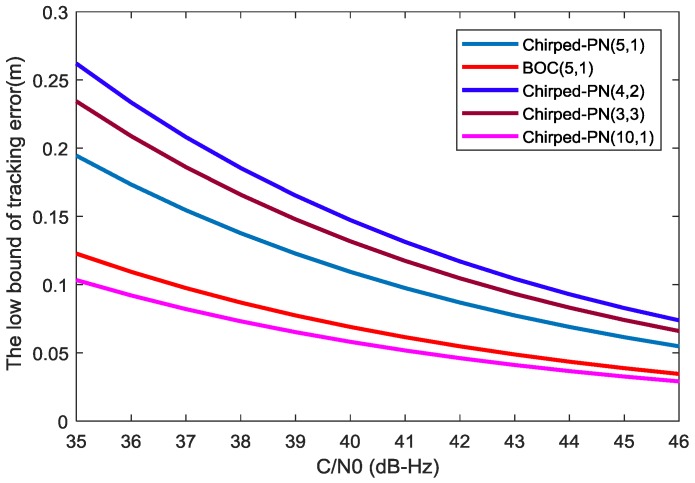
LBTEs for different modulations.

Based on (18), Figure 5 can be obtained with the following preconditions: (1) $B_{L}=0.5\;\mathrm{Hz}$, T=1 ms, $G_{w}\left(f\right)=N_{0}$, where $N_{0}$ is the PSD of thermal noise; (2) the same PDB of 30 MHz. Based on Figure 5, the following conclusions can be drawn. For the first situation, ChPN(5,1) owns the smallest LBTEs, ChPN(3,3) has smaller LBTEs than ChPN(4,2) due to there are more high-frequency components in the PDB of 30 MHz. For the second situation, BOC(5,1) has smaller LBTEs than ChPN(5,1). For the third situation, ChPN(10,1) has smaller LBTEs than ChPN(5,1). Furthermore, when the carrier-to-noise ratio (C/N0) increases, either the LBTE itself or the LBTE differences between ChPN signals with different sweep bandwidth or between ChPN and BOC become smaller.

**Figure 6:**
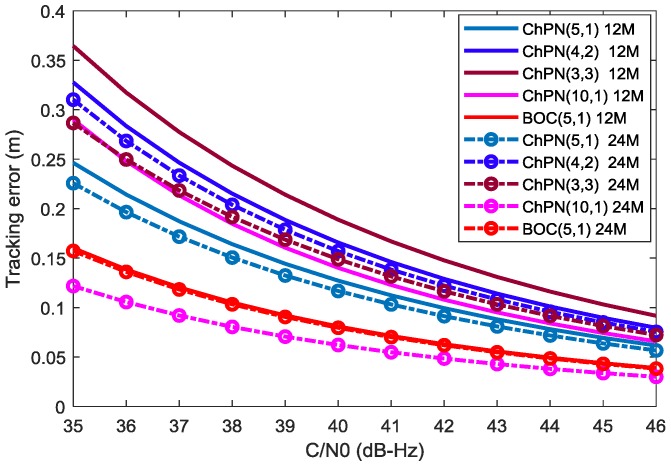
Tracking errors for ChPN(5,1), ChPN(4,2), ChPN(3,3), ChPN(10,1) and BOC(5,1).

Assuming non-coherent DLL is used, the tracking precision can be evaluated with the mathematical model presented by [26] as follows:

(19) σ=1TcBL∫−βr/2βr/2Gs(f)sin2(πfD)df(2π)2CN0(∫−βr/2βr/2fGs(f)sin(πfD)df)2×1+BL∫−βr/2βr/2Gs(f)cos2(πfD)dfTCN0(∫−βr/2βr/2fGs(f)cos(πfD)df)2(chip)

where *D* is the early-late spacing (ELS), the tracking precision curves of ChPN(5,1), ChPN(4,2), ChPN(3,3), ChPN(10,1) and BOC(5,1) are shown in Figure 6, which is drawn under the conditions that (1) code loop bandwidth is 0.5 Hz; (2) ELS is 5.86 m; and (3) coherent integration time is 1 ms. As we can see from the figure, when the C/N0 increases, either the tracking error itself or the tracking error differences between different signals become smaller. With the PDB increasing, the tracking error become smaller. For the first situation, ChPN(5,1) has the smallest tracking errors, ChPN(3,3) performs better than ChPN(4,2) with a PDB of 24 MHz, and worse than that with a PDB of 12 MHz. For the second situation, BOC(5,1) has a smaller tracking error than ChPN(5,1). For the third situation, ChPN(10,1) has a smaller tracking error than ChPN(5,1) with a PDB of 24 MHz; when the PDB is 12 MHz, however, the larger tracking error happens due to lots of frequency components are wasted which can be easily seen in Figure 4.

### 4.3. Multipath Resistance

**Figure 7:**
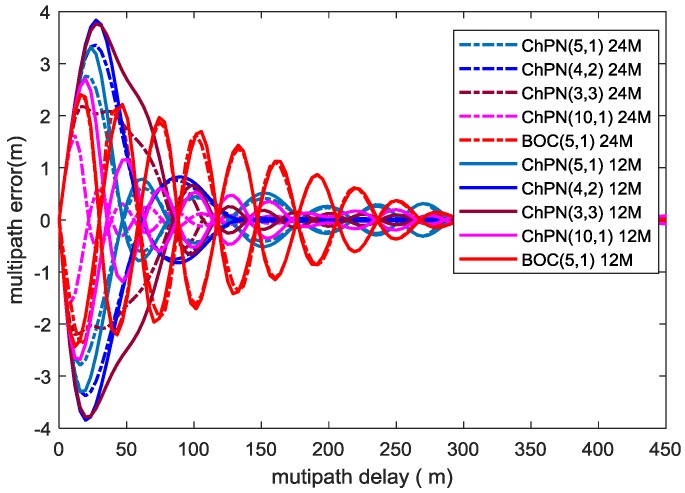
MPEEs vs multipath delay.

Tang developed an explicit expression for multipath error envelope (MPEE), which reflects the multipath mitigation performance of GNSS signals, expressed as follows [29]:

(20) $$\varepsilon \left(\tau \right)=\frac{\pm a\int_{-\beta_{r}/2}^{\beta_{r}/2}G_{s}\left(f\right)\sin\left(2\pi f\tau \right)\sin\left(\pi fD\right)df}{2\pi \int_{-\beta_{r}/2}^{\beta_{r}/2}fG_{s}\left(f\right)\sin\left(2\pi fD\right)\left[1\pm a\cos\left(2\pi f\tau \right)\right]df}\left(\mathrm{second}\right)$$

where τ is multipath delay, *a* is the amplitude ratio of multipath to direct path; when the phase difference between direct path and reflected path is 0 or 180 degrees, ‘±’ is ‘+’ or ‘−’, respectively. Based on (20), Figure 7 computes MPEEs of different modulations for a non-coherent delay DLL with an ELS of 5.86 m and a multipath to direct ratio (MDR) of −6 dB. As the figure shows, the users can achieve smaller MPEEs with a larger PDB. The MPEEs for multipath delay below 7 m and 5 m are practically equal for all the signals with PDBs of 24 MHz and 12 MHz, respectively. For the first situation, with a PDB of 12 MHz, ChPN(5,1) has the smallest MPEEs, followed by ChPN(4,2) and ChPN(3,3); when the PDB increase to 24 MHz, with a multipath delay less than 30 m, ChPN(3,3) has the smallest MPEEs followed by ChPN(5,1) and ChPN(4,2); ChPN(5,1) has the best overall multipath resistance performance. For the second situation, BOC(5,1) has smaller MPEEs than ChPN(5,1) when multipath delay below 40 m, MPEE curves of ChPN(5,1) converge much faster than that of BOC(5,1), ChPN(5,1) has a better overall multipath resistance. For the third situation, ChPN(10,1) is better than ChPN(5,1) in MPEEs.

**Figure 8:**
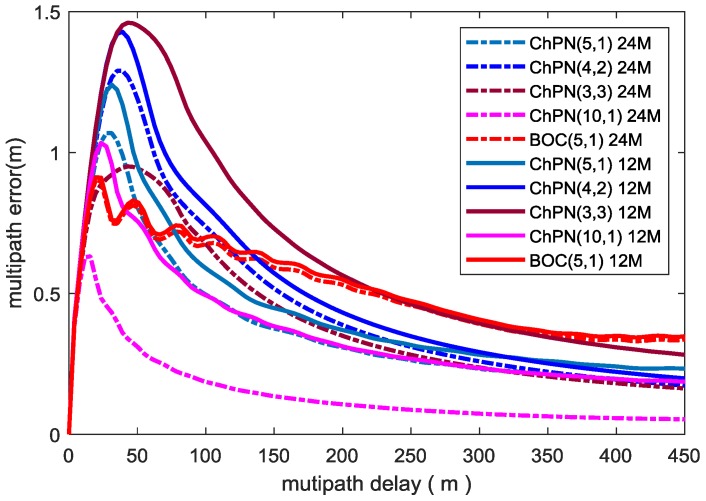
AWMEs vs multipath delay.

Figure 8 shows the average worst-case multipath errors (AWME) for various signals, which is drawn with the following model and the same conditions with Figure 7:

(21) $$\varepsilon_{a}=\frac{1}{\tau }\int_{0}^{\tau }\left[\frac{abs\left({\varepsilon \left(\tau \right)|}_{\varphi =0}\right)+abs\left({\varepsilon \left(\tau \right)|}_{\varphi =180^{o}}\right)}{2}\right]d\tau \left(\mathrm{second}\right)$$

where φ carrier phase difference of multipath and direct path. With the PDB increasing, the AWMEs become smaller. For the first situation, ChPN(5,1) has the smallest AWMEs followed by ChPN(4,2) and ChPN(3,3) with a PDB of 12 MHz; when the PDB is 24 MHz, ChPN(3,3) has the smallest AWMEs with a multipath delay below 50 m, ChPN(5,1) owns the smallest AWMEs if multipath delay exceeds 50 m, ChPN(4,2) performs worst over all multipath delay. For the second situation, BOC(5,1) has the smaller AWMEs than ChPN(5,1) with multipath delay less than 80 m and 50 m when the PDBs are 12 MHz and 24 MHz, respectively. For the third situation, ChPN(10,1) has smaller AWMEs than ChPN(5,1). Moreover, due to a PDB of 12 MHz is not sufficient to include the frequency components of ChPN(10,1), the AWME curves of ChPN(10,1) “jump” from a high level to low level when the PDB changes from 12 MHz to 24 MHz.

### 4.4. ACF Peaks Ambiguity

Due to the similar modulation principle to BOC, ChPN also suffers ACF peaks ambiguity, which is caused by the ACF side-peaks. That phenomenon makes it difficult for receivers to acquire and track the signal correctly, and sometimes false locking occurs in the presence of thermal noise or dynamic stress. Therefore, it is necessary for us to present the MSPRs for ChPN signals with different *p* and *q*.

The time delay corresponding to the maximum-side-peak can be computed by the following implicit equation:

(22) $$\tau_{s}=\arg\min\left\{\frac{dR\left(\tau \right)}{d\tau }=0,\tau >0\right\}$$

then, substituting $\tau_{s}$ into (9), we obtain auto-correlation value *R*_m_, the MSPR obtained by computing the following equation:

(23) $$MSPR=20\log_{10}\left(R_{m}\right)$$

**Figure 9:**
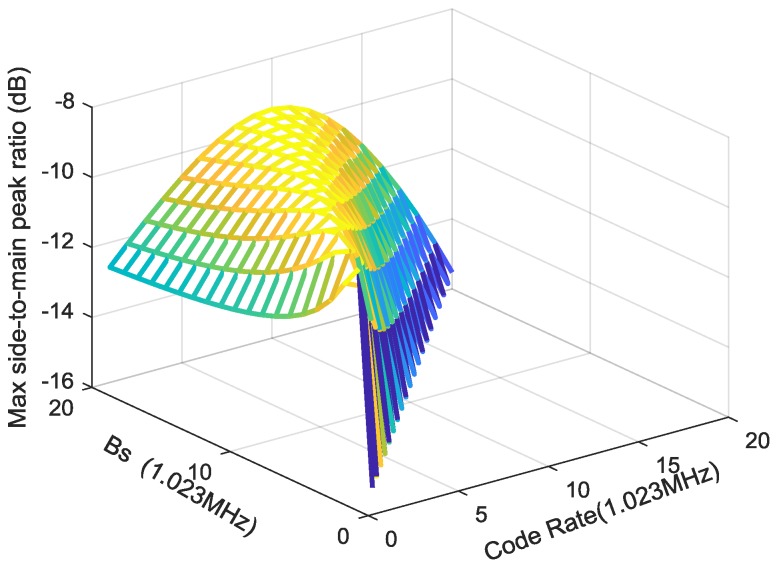
MSPRs VS various code rates and sweep bandwidths.

Figure 9 is drawn based on (23), which presents the MSPRs of various ChPN modulations. As shown in the figure, the MSPRs reaches the minimum amplitude when the code rate equals to the sweep bandwidth and reaches the maximum amplitude when the sweep bandwidth is double of the code rate. The figure gives us a reference when we choose parameters for ChPN signals.

### 4.5. Cross-Correlation Comparison

**Figure 10:**
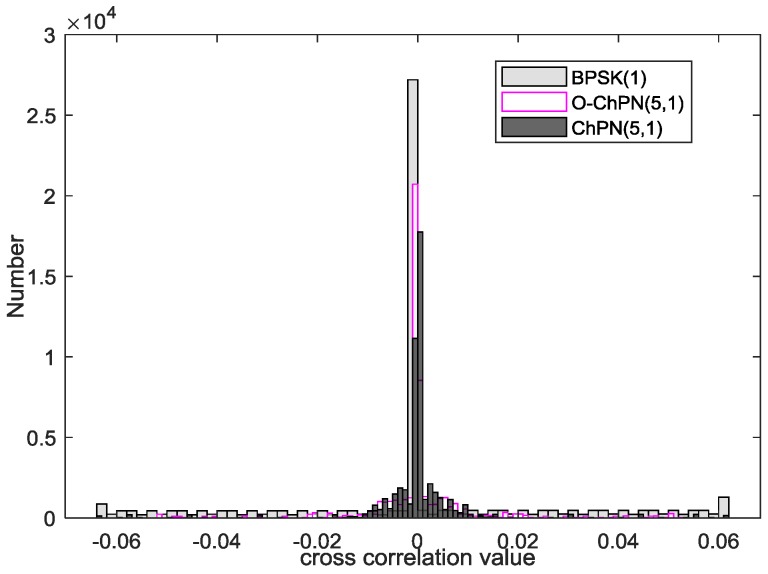
Cross correlation analysis.

As we know, the cross correlation is an essential indicator for a ground-based navigation system, which reflects the resistance to near-far effects. The following statistic histogram is drawn with the signals that modulated by the C/A code of satellite 1 and 2. The cross-correlation amplitudes are normalized with the value of auto-correlation peak. As we can see from Figure 10, BPSK(1) and ChPN(5,1) have the same maximum cross correlation, which is larger than O-ChPN(5,1). Furthermore, most samples of ChPN(5,1) and O-ChPN(5,1) are located in −0.02 to 0.02, while BPSK(1) contains more samples in higher range, such as 0.02 to 0.06. The auto-to-cross-correlation peak ratio (ACCR) of BPSK(1) and ChPN(5,1) is 24 dB, while the ACCR of O-ChPN(5,1) is about 26 dB, 2 dB better than the above two.

## 5. Simulation

**Figure 11:**
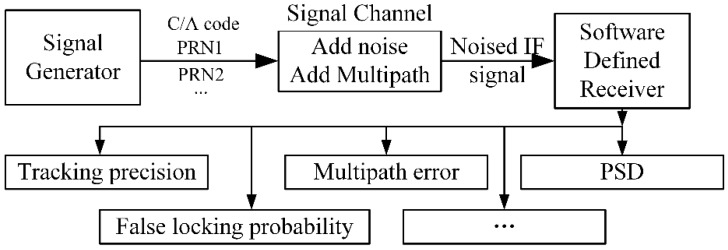
Simulation method.

In order to explore the signal performance, the computer simulation is performed, with the method shown Figure 11. First, the digital IF navigation signal is generated by MATLAB, and saved in a disk. Second, we load this signal samples, add noise and multipath signal to the original samples with a specified CN0 and MDR when we use. Then, the signal samples are processed by a software defined receiver (SDR). Finally, the signal performance is evaluated by the tracking results output from SDR.

**Figure 12:**
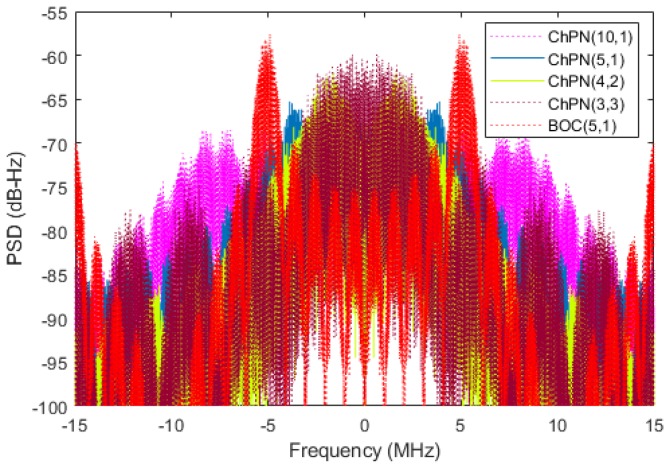
Simulated PSD of various signal.

Figure 12 shows the simulated PSDs of ChPN(5,1), ChPN(4,2), ChPN(3,3), ChPN(10,1) and BOC(5,1), which quite match with Figure 4. According to Figure 12, a signal with a wider sweep bandwidth owns a flatter PSD; the signals of situation 1 differ in characteristics, ChPN(3,3) is similar to BPSK, ChPN(4,2) is similar to BOC, and ChPN(5,1) is similar to chirp; for situation 2, ChPN(5,1) has a more flatter PSD than BOC(5,1) while BOC(5,1) has more high-frequency components; for situation 3, ChPN(10,1) has a flatter and wider PSD than ChPN(5,1).

**Figure 13:**
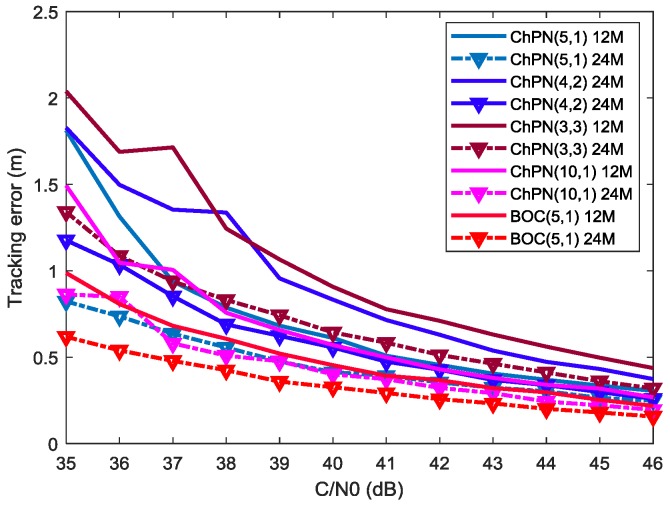
Simulated tracking errors of various signals with bandwidth of 12 MHz and 24 MHz.

Figure 13 shows the simulated tracking errors of different signals, which is drawn with an ELS of 5.86 m, a sampling rate of 61 MHz, a code loop bandwidth of 0.5 Hz, and a coherent integration time of 1 ms. According to the figure, we can draw some conclusions: (1) either the tracking error or the tracking error difference between different signals decrease when the C/N0 increase; (2) the tracking errors become smaller when the PDB increase; (3) a wider sweep bandwidth leads to better tracking performance for the signals that own the same or different MLBs; (4) BOC(5,1) owns smaller tracking errors than ChPN(5,1) with the same MLB.

As we can see from Figure 6 and Figure 13, the two figures are similar in variation trend, but differ in numerical value. Figure 6 is obtained by calculating with a mathematical model. As some factors that impact the tracking errors are not considered in that model but encountered in SDR, such as carrier phase differences, carrier frequency differences and sampling frequency.

**Figure 14:**
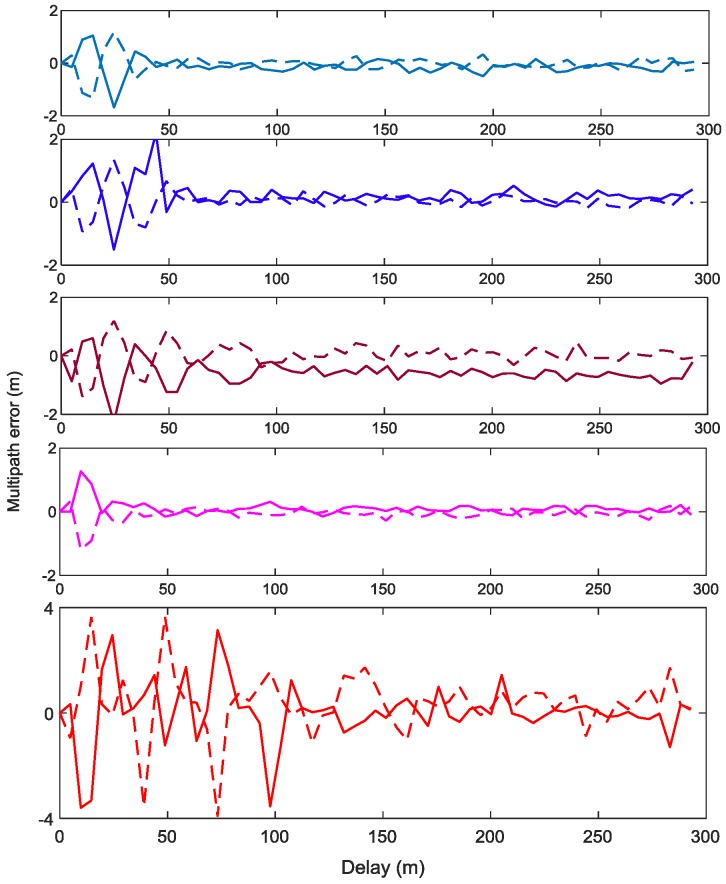
Simulated multipath errors for ChPN(5,1), ChPN(4,2), ChPN(3,3), ChPN(10,1), and BOC(5,1) from top to down. The dash lines indicate the out of phase multipath, the solid lines indicate the in phase multipath.

**Figure 15:**
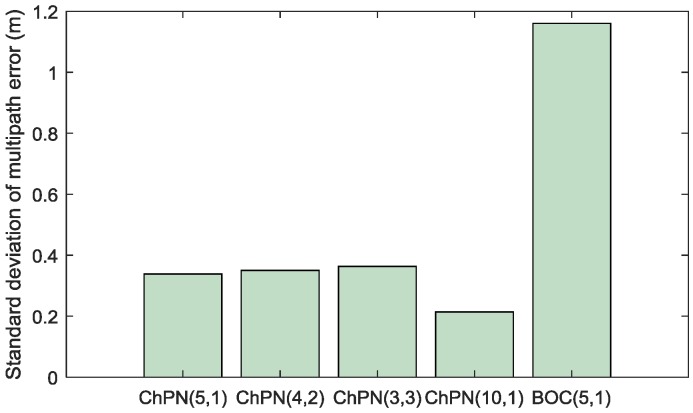
Standard deviation of multipath error.

MPEE reflects the maximum ranging error caused by one-ray multipath with specific parameters, which is the theory limit calculated by mathematical model. Hence, it is hard for us to obtain MPEEs from a SDR, but multipath errors are reachable. Figure 14 shows the simulated multipath errors for various signals, which is drawn under the condition that C/N0 is 40 dB-Hz, ELS is 5.86 m, sampling rate is 61 MHz, the code loop bandwidth is 0.5 Hz, MDR is −6 dB, PDB is 24 MHz. According to Figure 14 and Figure 15, some conclusions can be drawn. First, for situation 1, the signal with a wider sweep bandwidth has a smaller multipath error among the alternative signals, such as ChPN(5,1) has the smallest multipath error and the fastest convergence speed followed by ChPN(4,2) and ChPN(3,3). Secondly, for situation 2, ChPN(5,1) has a smaller multipath error than BOC(5,1), the convergence speed is faster than that of BOC(5,1). Thirdly, for situation 3, ChPN(10,1) has a smaller multipath error and a faster convergence speed than ChPN(5,1). The variation trends are similar to Figure 7, as well as the conclusions.

Figure 15 shows the standard deviation of multipath error of various signals, which reflects the jitter of multipath error. ChPN(5,1) has the smallest multipath error standard deviation among the signals of situation 1. Both ChPN(5,1) and ChPN(10,1) has the smaller multipath error standard deviation than BOC(5,1).

**Figure 16:**
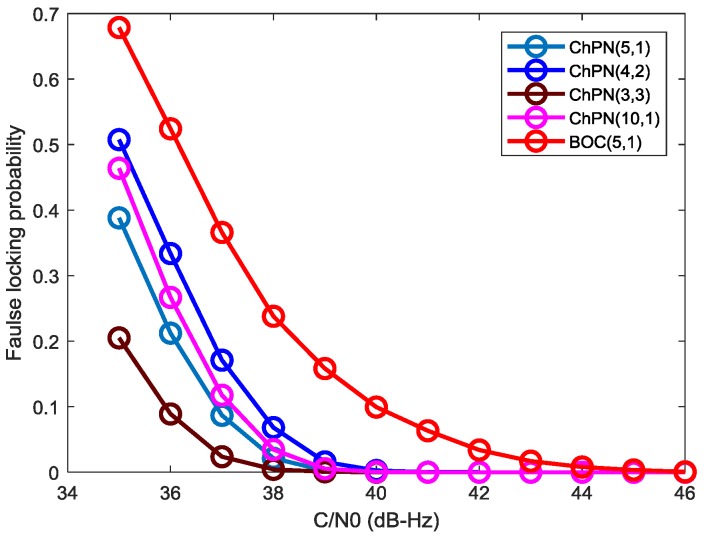
False locking probability caused by side-peaks for various signals.

In order to explore the ACF peaks ambiguity of the signals, we carry out the Monte Carlo simulation with 10,000 runs. Figure 16 shows the simulated false locking probability (FLP) caused by ACF side-peaks, which is drawn with a PDB of 24 MHz, a sampling rate of 61 MHz, and a coherent integration time of 1 ms. Generally, the FLP become smaller when the C/N0 increase. The FLP of BOC(5,1) is larger than ChPN(5,1) due to its large MSPR and numerous side-peaks as shown in Figure 3. Owing to the smallest side-peak amplitude, as Figure 3 and Figure 9 show, ChPN(3,3) owns the smallest FLP among the ChPN signals with a MLB of $12f_{0}$, i.e., ChPN(3,3), ChPN(4,2) and ChPN(5,1). Furthermore, ChPN(10,1) has a slightly larger FLP than ChPN(5,1) due to the similar MSPR and the more side-peaks, as Figure 3 and Figure 9 show. In summary, the side-peaks number and MSPR are the two factors that impact the ACF peaks ambiguity, but MSPR is the major factor.

**Figure 17:**
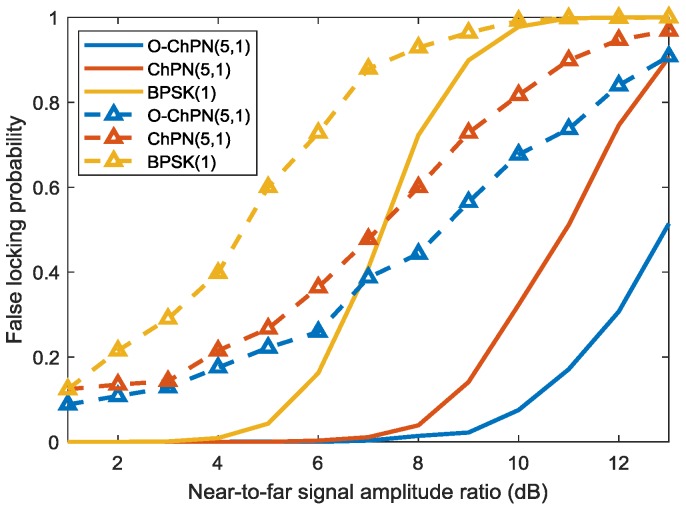
False locking probability with different near-to-far signal amplitude ratio. The dash and the solid lines are drawn with the C/N0 of 40 dB and 45 dB, respectively.

Near-far effect is caused by the cross correlation of the signals that are coming from the transmitters of different distances, which will affect the receiver to track the correct but weaker navigation signal. The Monte Carlo simulation is carried out with a PDB of 24 MHz, a sampling rate of 61 MHz, and a coherent integration time of 1 ms to explore the anti-near-far effect performance of the proposed signals. In the simulation, the signal with the C/A code of GPS satellite 1 is acting as the “far” signal; the signal with the C/A code of GPS satellite 2 is acting as the “near” signal, which is treated as the “interference”. As Figure 17 shows, the signal with a larger C/N0 has a smaller FLP; as near-to-far signal amplitude ratio increase, the FLP become larger; the O-ChPN has the best anti-near-far performance, followed by ChPN and BPSK. O-ChPN can be used in a system that do not need much transmitters or the bandwidth is sufficient, such as the ISM band.

## 6. Conclusions

Based on the chirp technique widely applied in the fields of communication, radar, and indoor navigation, we have proposed a high-accuracy, low-ambiguity, low-power consumption signal modulation method named ChPN. The signal model, ACF, and PSD have been presented, and the signal generation and detection methods have been discussed. The O-ChPN is also discussed. The theoretical performance of ChPN signals are analyzed, the simulation is also carried out to evaluate the signal performance.

The signals are analyzed in three situations: (1) the ChPN signals with the same MLB and different *p* and *q*, such as ChPN(5,1), ChPN(4,2) and ChPN(3,3); (2) the different signals with the same MLB, such as ChPN(5,1) and BOC(5,1); (3) the ChPN signals with different MLBs, such as ChPN(5,1) and ChPN(10,1). By analyzing the ACF, PSD, LBTE, tracking error, MPEE, AWME and ACF peaks ambiguity characteristics of ChPN and BOC signals, we have found that: (1) for the first situation, ChPN(5,1) has the best tracking precision and multipath resistance, while ChPN(3,3) has the smallest MSPR and FLP; (2) for the second situation, BOC(5,1) has a better tracking precision and LBTEs, while ChPN(5,1) provides better overall resistance to multipath effects and has smaller MSPR and FLP; (3) for the third situation, ChPN(10,1) has better tracking precision, better multipath resistance, while ChPN(5,1) has smaller FLP. O-ChPN can mitigate the near-far effect, although it may decrease the transmitter amount. Last but not least, ChPN receivers can be implemented by minor modification of GNSS receivers owing to their similar signal structures. In summary, ChPN is a feasible and promising signal modulation method for PRN code ranging systems.

## Figures and Tables

**Table 1:** Analysis situations.

Situations	Signals	Characteristics
1	ChPN(5,1), ChPN(4,2), ChPN(3,3)	ChPN signals with the same MLB, different sweep bandwidths.
2	ChPN(5,1), BOC(5,1)	Different waveforms with the same code rate and MLB.
3	ChPN(10,1), ChPN(5,1)	ChPN signals with the same code rate and different MLBs.

## Appendix A

The spectrum of chirp signal with a sweep bandwidth of *B_sw_* and a chip duration of *T_c_* can be expressed as [23]:

(A1) $$\begin{array}{l} P\left(\omega \right)=\int_{-T_{c}/2}^{T_{c}/2}\sqrt{2}\cos\left(\omega_{c}t+\frac{\mu t^{2}}{2}\right)\exp\left(-j\omega t\right)dt\\ =\frac{\sqrt{2}}{2}\int_{-T_{c}/2}^{T_{c}/2}\exp\left[j\left(\omega_{c}t-\omega t\right)+j\frac{\mu t^{2}}{2}\right]dt+\frac{\sqrt{2}}{2}\int_{-T_{c}/2}^{T_{c}/2}\exp\left[-j\left(\omega_{c}t+\omega t\right)-j\frac{\mu t^{2}}{2}\right]dt \end{array}$$

where $\mu =B_{sw}/T_{c}$, $\omega_{c}=2\pi f_{c}$, $B_{sw}=2\pi B_{s}$. Here, $\sqrt{2}$ is used to ensure the power is 1 (unity power). As we can see P(ω) can be divided into two terms.

### A.1. The First Term

The first term of (A1) can be expressed as [23]:

(A2) $$U_{1}\left(f\right)=\frac{\sqrt{2}}{2}\sqrt{\frac{T_{c}}{2B_{s}}}\exp\left[-j\frac{T_{c}\pi {\left(f-f_{c}\right)}^{2}}{B_{s}}\right]\left[C\left(X_{1}\right)+jS\left(X_{1}\right)+C\left(X_{2}\right)+jS\left(X_{2}\right)\right]$$

where ω, $\omega_{c}$, $B_{sw}$ are replaced by 2πf, $2\pi f_{c}$, $2\pi B_{s}$, respectively; and:

(A3) $$\{\begin{array}{c} C\left(X\right)=\int_{0}^{X}\cos\frac{\pi y^{2}}{2}dy\\ S\left(X\right)=\int_{0}^{X}\sin\frac{\pi y^{2}}{2}dy \end{array}$$

are the Fresnel cos and sin integrals, the *X*1 and *X*2 are expressed as:

(A4) $$\{\begin{array}{c} X_{1}=\frac{B_{s}+2\left(f-f_{0}\right)}{\sqrt{2B_{s}/T_{c}}}\\ X_{2}=\frac{B_{s}-2\left(f-f_{0}\right)}{\sqrt{2B_{s}/T_{c}}} \end{array}$$

### A.2. The Second Term

The second term is:

(A5) $$U_{2}\left(\omega \right)=\frac{\sqrt{2}}{2}\int_{-T_{c}/2}^{T_{c}/2}\exp\left[-j\left\{\left(\omega_{c}+\omega \right)t+\frac{\mu t^{2}}{2}\right\}\right]dt$$

which can be expressed as:

(A6) $$U_{2}\left(\omega \right)=\frac{\sqrt{2}}{2}\exp\left[j\frac{{\left(\omega +\omega_{c}\right)}^{2}}{2\mu }\right]\int_{-T_{c}/2}^{T_{c}/2}\exp\left[-j\frac{\mu }{2}{\left(t+\frac{\left(\omega +\omega_{c}\right)}{\mu }\right)}^{2}\right]dt$$

with the assumption of $\sqrt{\mu }\left(t+\frac{\omega +\omega_{0}}{2}\right)=\sqrt{\pi }x$, and then $dt=\sqrt{\pi /\mu }dx$, (A6) can be expressed as:

(A7) $$U_{2}\left(\omega \right)=\frac{\sqrt{2}}{2}\sqrt{\frac{\pi }{\mu }}\exp\left[j\frac{{\left(\omega +\omega_{c}\right)}^{2}}{2\mu }\right]\int_{-X_{3}}^{X_{4}}\exp\left[-j\frac{\pi }{2}x^{2}\right]dx$$

*X*_3_ and *X*_4_ are as:

(A8) {X3=μTc2−(ω+ωc)πμX4=μTc2+(ω+ωc)πμ

Based on the Euler formula:

(A9) $$\exp\left[-j\frac{\pi }{2}x^{2}\right]=\cos\left(\frac{\pi }{2}x^{2}\right)-j\sin\left(\frac{\pi }{2}x^{2}\right)$$

(A7) can be re-written as:

(A10) $$U_{2}\left(\omega \right)=\frac{\sqrt{2}}{2}\sqrt{\frac{\pi }{\mu }}\exp\left[j\frac{{\left(\omega +\omega_{c}\right)}^{2}}{2\mu }\right]\int_{-X_{3}}^{X_{4}}\left[\cos\left(\frac{\pi }{2}x^{2}\right)-j\sin\left(\frac{\pi }{2}x^{2}\right)\right]dx$$

which can be further simplified as:

(A11) $$U_{2}\left(\omega \right)=\frac{\sqrt{2}}{2}\sqrt{\frac{\pi }{\mu }}\exp\left[j\frac{{\left(\omega +\omega_{c}\right)}^{2}}{2\mu }\right]\left\{\int_{-X_{3}}^{X_{4}}\cos\left(\frac{\pi }{2}x^{2}\right)dx-j\int_{-X_{3}}^{X_{4}}\sin\left(\frac{\pi }{2}x^{2}\right)dx\right\}$$

Substituting Fresnel cosine and sine function into the above equation, we have:

(A12) $$U_{2}\left(\omega \right)=\frac{\sqrt{2}}{2}\sqrt{\frac{\pi }{\mu }}\exp\left[j\frac{{\left(\omega +\omega_{c}\right)}^{2}}{2\mu }\right]\left[C\left(X_{3}\right)+C\left(X_{4}\right)-jS\left(X_{3}\right)-jS\left(X_{4}\right)\right]$$

C(X) and S(X) are shown in (A3). Substituting ω=2πf, $\omega_{c}=2\pi f_{c}$, $\mu =B_{sw}/T_{c}$, $B_{sw}=2\pi B_{s}$ into (A12), then we have:

(A13) $$U_{2}\left(f\right)=\frac{1}{2}\sqrt{\frac{T_{c}}{B_{s}}}\exp\left[j\frac{T_{c}\pi {\left(f+f_{0}\right)}^{2}}{B_{s}}\right]\left[C\left(X_{3}\right)+C\left(X_{4}\right)-jS\left(X_{3}\right)-jS\left(X_{4}\right)\right]$$

where:

(A14) $$\{\begin{array}{c} X_{3}=\frac{B_{s}-2\left(f+f_{c}\right)}{\sqrt{2B_{s}/T_{c}}}\\ X_{4}=\frac{B_{s}+2\left(f+f_{c}\right)}{\sqrt{2B_{s}/T_{c}}} \end{array}$$

### A.3. The Expression

According to (A2) and (A13), the P(f) is shown in the following expression:

(A15) $$P\left(f\right)=U_{1}\left(f\right)+U_{2}\left(f\right)$$

which can be also expressed as:

(A16) $$P\left(f\right)=\frac{1}{2}\sqrt{\frac{T_{c}}{B_{s}}}\left\{\begin{array}{l} \exp\left[-j\frac{T_{c}\pi {\left(f-f_{c}\right)}^{2}}{B_{s}}\right]\\ \times \left[C\left(X_{1}\right)+jS\left(X_{1}\right)+C\left(X_{2}\right)+jS\left(X_{2}\right)\right]\\ +\exp\left[j\frac{T_{c}\pi {\left(f+f_{c}\right)}^{2}}{B_{s}}\right]\\ \times \left[C\left(X_{3}\right)+C\left(X_{4}\right)-jS\left(X_{3}\right)-jS\left(X_{4}\right)\right] \end{array}\right\}$$

which has been shown in (12) previously.
